# Simulating optical coherence tomography for observing nerve activity: A finite difference time domain bi-dimensional model

**DOI:** 10.1371/journal.pone.0200392

**Published:** 2018-07-10

**Authors:** Francesca Troiani, Konstantin Nikolic, Timothy G. Constandinou

**Affiliations:** Centre for Bio-Inspired Technology, Imperial College London, London, United Kingdom; Bascom Palmer Eye Institute, UNITED STATES

## Abstract

We present a finite difference time domain (FDTD) model for computation of A line scans in time domain optical coherence tomography (OCT). The OCT output signal is created using two different simulations for the reference and sample arms, with a successive computation of the interference signal with external software. In this paper we present the model applied to two different samples: a glass rod filled with water-sucrose solution at different concentrations and a peripheral nerve. This work aims to understand to what extent time domain OCT can be used for non-invasive, direct optical monitoring of peripheral nerve activity.

## 1 Introduction

Optical coherence tomography is a low coherence interferometric technique that has been first used in 1991 to examine the peripapillary region of the retina [1] and has, since then, played a very important role in medical imaging. There are different techniques available to simulate a process like OCT, e.g. Monte Carlo [2, 3] or computational electrodynamics techniques. Monte Carlo techniques rely on random sampling and can, in principle, be used to solve any problems having a probabilistic interpretation. Computational electrodynamics includes all the techniques that model, through approximations of Maxwell equations, the interaction of electromagnetic waves with physical objects and the environment. They work best when the wavelength of the electromagnetic wave considered is comparable with the smallest detail of the studied object. Examples of computational electrodynamics techniques are the method of moments and the finite element method in the frequency domain and the finite-difference time-domain (FDTD) method in the time domain. For the interested reader, we recommend reading references [4, 5]. We have decided to use the FDTD method as its time domain nature allows to obtain results for a range of frequencies using a single simulation. This allows to simulate the low coherence gate property of OCT by using, as the light source, a pulse which length in time is chosen to match the desired width of the frequency spectrum.

To this day there are several FDTD models for OCT imaging, and the ones currently known in literature focus mostly on trying to simulate big or three dimensional environments in a reasonable (38 minutes using 6 cores of an Intel Xeon E5645 CPU for a bi dimensional simulation [6] and 17 hours using 8 cores of a dual Intel Xeon E5-2650 v2 for a three dimensional one [7]) time. They achieve this results by increasing the dimension of the FDTD cell, thus lacking of enough spatial resolution to simulate small scatterers in biological tissues. The aim of this model is to obtain A-scans in simulation domains containing scatterers with diameters comparable to the wavelength while maintaining a reasonable computational time (7 hours using one single core of an Intel^®^ Xeon^®^ X5680 CPU).

### 1.1 OCT for neural recording

During the years, different techniques have been developed to record neural activity, varying in the level of invasiveness. Non invasive techniques—e.g. EEG (Electro EncephaloGraphy, monitoring brain activity by recording electric signals from the surface of the scalp) or fMRI (functional Magnetic Resonance Imaging, measuring brain activity by detecting associated changes in blood flow)—measure activity at a global level. On the other hand, invasive techniques—e.g. penetrating micro electrodes—are capable of a much higher spatial resolution. At this point in time, it is necessary to establish techniques that can give the best of the two worlds, providing both high resolution and low invasiveness. Optical recordings have been gaining momentum in the past 50 years and the advent of calcium sensitive dyes and genetically encoded indicators has revolutionised neural recordings. However both techniques exhibit either phototoxicity or very low brightness and this limits their usage in *in vivo* experiments.

Optical recordings for neural activity started in the 1950s [8] and proceeded into the late ‘60s when it was showed that change in forward scattered light from a nerve bundle during activity is of the order of one to ten parts per million and that the optical effect lasts for roughly a millisecond [9, 10]. In more recent years, scientists have been focussing on ways to obtain information on neural activity by observing changes in intrinsic optical properties of the neurons. The use of OCT has already been proved useful for structural [11–13] and functional [14–16] in brain tissue and—by using phase sensitive or polarization sensitive OCT—in peripheral nerves [17–20]. We are working on using time domain OCT to detect compound action potential in a peripheral nerve. The focus on time domain relates to our ultimate goal of developing a miniaturised low cost OCT device, however this model can be applied without no further intrinsic modifications to spectral domain systems. The methods and simulation tool described herein has been developed to gain insights to this specific application.

## 2 Materials and methods

The FDTD method is one of the simplest of the computational electrodynamics techniques and it can solve a broad range of problems in a very accurate way. It was first developed by Yee [21] in 1966, when the main applications of electromagnetic simulations were in the defence field, and from 1990 the interest in this technique has expanded to many different areas [22] including electromagnetic field interactions with biological media [23–25].

FDTD is a grid-based differential numerical modelling method where both the spatial and temporal derivatives that appear in Maxwell’s equations are discretised using a central-difference approximation. Central-difference approximations exist in several orders, this work uses a second order one:

$${\frac{df\left(x\right)}{dx}|}_{x=x_{0}}\begin{array}{c} \approx \frac{f\left(x_{0}+\frac{h}{2}\right)-f\left(x_{0}-\frac{h}{2}\right)}{h}. \end{array}$$ (1)

It has to be noted that this approach provides an approximation of the value of the derivative of the function at *x*_0_ but the function is sampled at the neighbouring points *x*_0_ + *h* and *x*_0_ − *h*.

**Fig 1:**
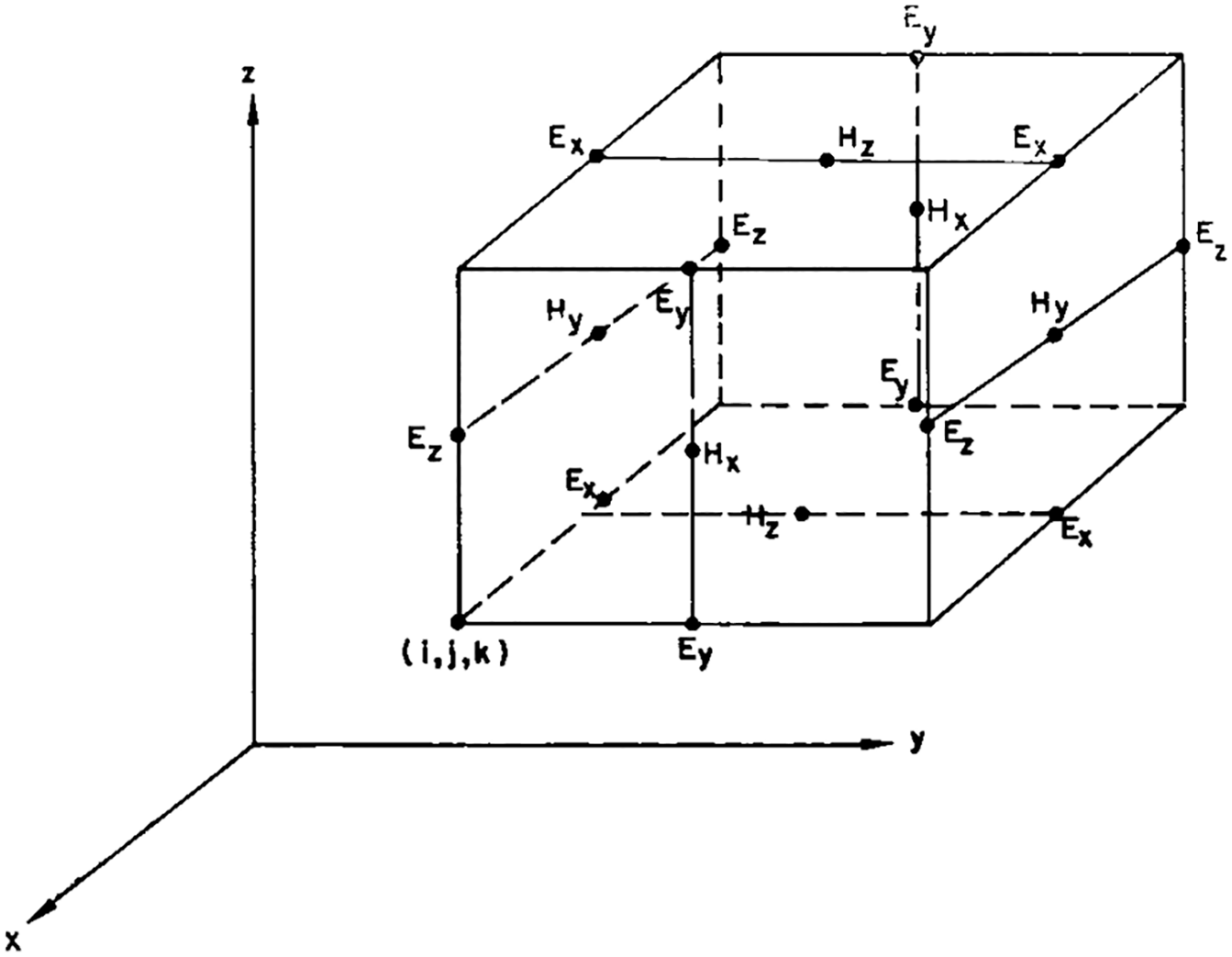
Yee lattice with positions of various field components. The components of the electric field are in the middle of the edges and the components of the magnetic fields are in the centre of the faces. ©1966 IEEE. Reprinted, with permission, from [21].

The algorithm used in this work is the Yee algorithm (Fig 1 shows a representation of Yee lattice) [21]. Despite being the first version of FDTD, this algorithm is very robust and of easy implementation and it can be summarised as follows:

1. Space and time are discretised—for each point in space and time (*i*, *j*, *k*, *t*) = (*i*Δ*x*, *j*Δ*y*, *k*Δ*z*, *q*Δ*t*)—and the derivatives in Ampere’s and Faraday’s laws are replaced with finite differences.
2. A new set of “updated equations” expressing the new fields in term of the past ones is obtained solving the difference equations.
3. The magnetic field is evaluated.
4. The electric field is evaluated.
5. The previous three steps are repeated until the end of the simulation is reached.

This algorithm can be applied to one, two and three-dimensional problems. The simulations presented in this paper are bi-dimensional as both the samples considered present cylindrical symmetry and the region of interest lays in their cross section.

**Fig 2:**
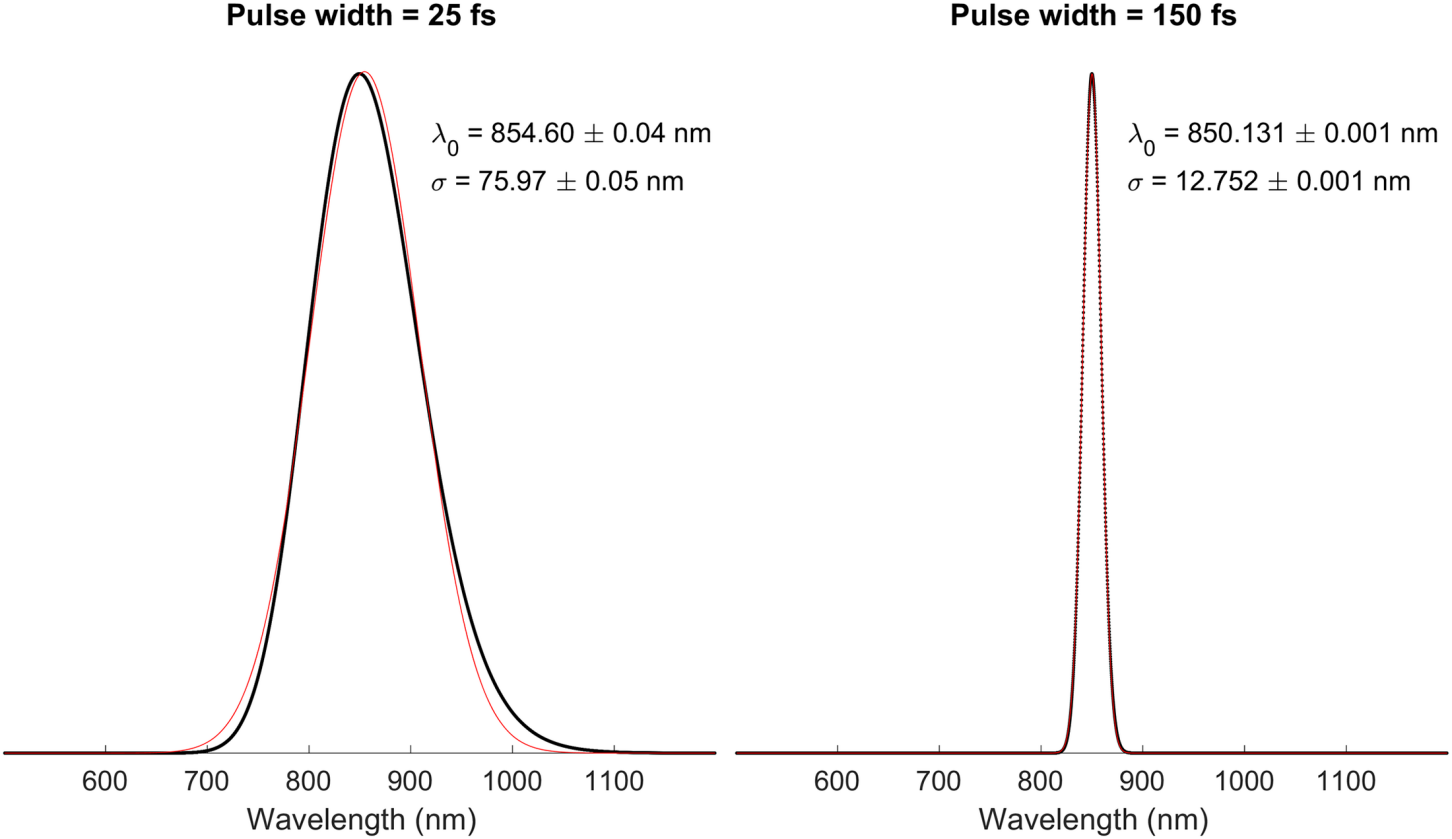
Frequency spectrum for pulses of different lengths, fitted with a Gaussian curve. In black the simulated pulse, in red the Gaussian fit. It is possible to notice that the broader spectrum is not a perfect Gaussian; this is due to the numerical dispersion being more pronounced in a source with a bigger frequency range.

As previously mentioned, the broadband spectrum of an OCT light source can be modelled in an FDTD simulation by changing the length of the pulsed used. Fig 2 shows the spectra obtained from pulses with a width of 25 fs and 150 fs and reported as a function of the wavelength.

For FDTD to be reliable and converge, the discretisation of the space needs to be at least an order of magnitude smaller than the wavelength. This means that at visible and near infra-red wavelengths, there is a limit on the dimension of the domain for the simulation to be carried out in a reasonable amount of time. Moreover, the temporal and spatial steps have to be in a relation such as the Courant number $S_{c}=\frac{c\Delta t}{\Delta x}\leq \frac{1}{\sqrt{D}}$, where c is the light speed in vacuum and D is the number of dimensions of the simulation.

## 3 The model

Without loss of generality, the field is assumed to be polarised in the x-direction and presents a Gaussian shape.

The simulations reported in this paper have been run with both pulse lengths shown in Fig 2. These two specific pulse lengths have been chosen to represent a super luminescent diode (SLD) with a broadness of *σ* ≃ 76 nm, which is quite common in the OCT community, and one with *σ* ≃ 13 nm, which corresponds to what can be typically found in low cost SLDs.

Two different samples have been studied using this computational model: a glass rod filled with sucrose solutions at different concentration and part of a myelinated nerve made of one single fascicles of axons (e.g. *Xenopus Laevis*’ sciatic nerve, the specimen we are going to use in our upcoming experiments). In both cases the refractive indexes of the different layers of the simulation domain are considered to be constant over the range of frequencies considered, as the dispersion in water for the narrow range of frequency considered can be assumed insignificant [26] and would just add computational complexity and time to the simulations. Therefore the only dispersion in the simulations is the numerical one which is due to the discrete nature of the FDTD grid.

### 3.1 Glass rod model

**Fig 3:**
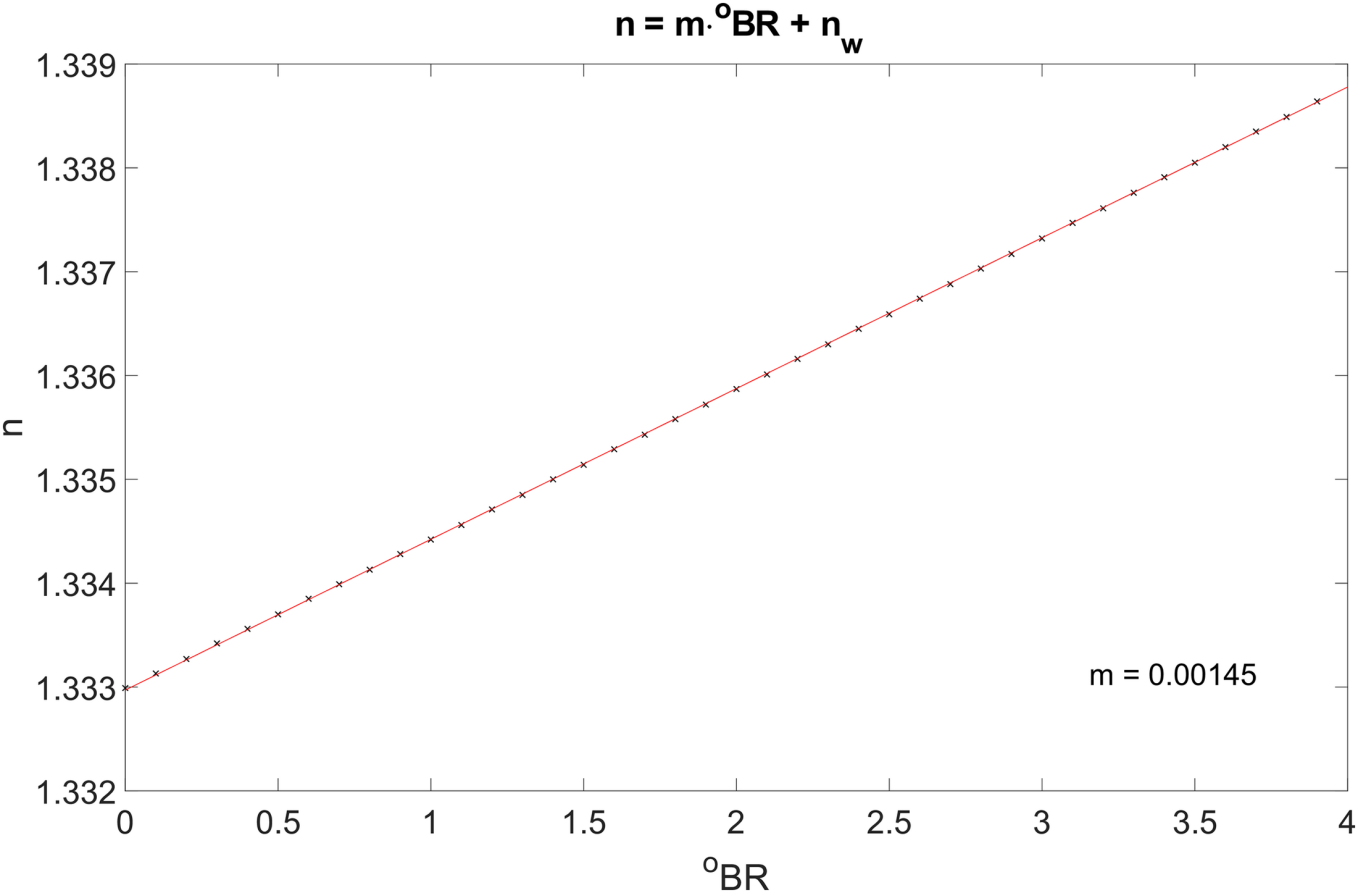
Refractive index of a water-sucrose solution as a function of the sucrose concentration at 589.3 nm [27]. In this work we are assuming that it is possible to translate the data to obtain values for different wavelengths.

This model has been developed to obtain a result that could be easily verified experimentally. By changing the concentration of the water-sucrose solution inside the rods it is possible to obtain different values for its refractive index which can be computed using the BRIX scale [27] (one degree Brix is defined as 1 g of sucrose dissolved in 100 g of water). Fig 3 shows that, at low concentrations, the refractive index of the solution is directly proportional to the concentration of sugar in the solution. The data used to obtain this value have been obtained for a wavelength of 589.3 nm and it has been assumed that to obtain the data for a different wavelength it is possible to translate rigidly the curve so that the intercept corresponds to the refractive index of water at the chosen wavelength. In the case of λ = 850 nm, this results in the curve becoming *n* = *m*⋅^*o*^ *BR* + *n*_*w*@850*nm*_, where *m* = 0.00145 and *n*_*w*@850*nm*_ = 1.3290.

The simulation environment consists of three layers: water (*n*_*w*@850*nm*_ = *n* = 1.3272), glass (*n*_*w*@850*nm*_ = 1.5098) and sucrose solution.

### 3.2 Nerve model

**Fig 4:**
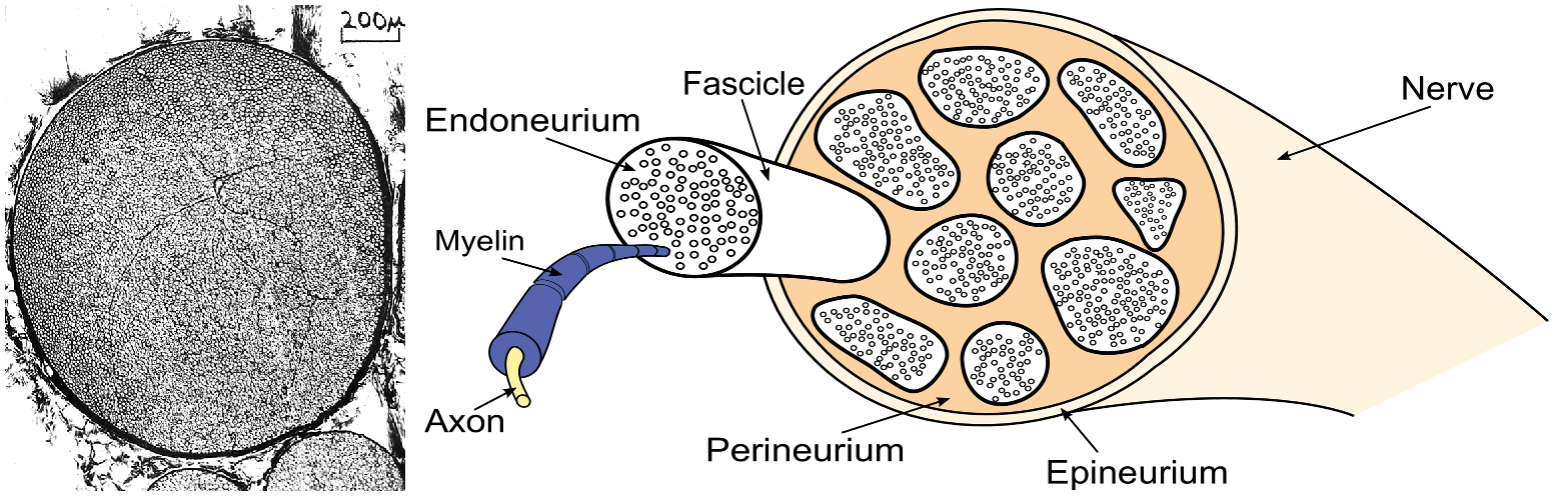
Peripheral nerve. Right: schematic structure of a nerve. Left: *Xenopus Laevis*’ sciatic nerve cross section [28]. It is possible to see how this nerve is made of a single bundle of axons surrounded by the perineum and loose epineurium.

The right panel of Fig 4 shows the structure of a nerve and its different layers, while the left panel shows a cross section of the nerve considered for this study, the *Xenopus Laevis*’ sciatic nerve [28]. This specific nerve is widely used in neuroscientific applications due to being made of one single fascicle, property that allows for a clearer signal in both the electrical and, considering the lack of multiple interfaces between different fascicles, the optical measurements and is the specimen we will be using for our future experiments.

**Fig 5:**
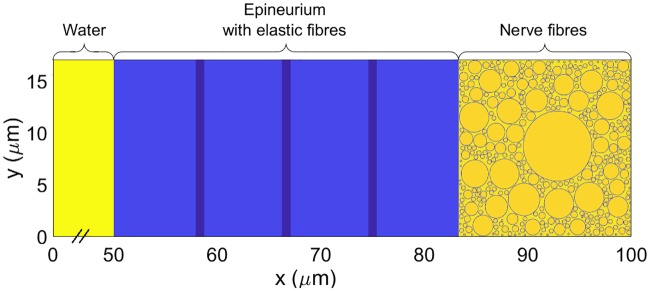
Simulation domain for the nerve model.

Fig 5 shows the simulation domain considered for the nerve model. It is a domain of 100 × 17 *μ*m^2^ of which the first half is made of Ringer’s solution and the second of the nerve and its fibres. In the *Xenopus Laevis*’ sciatic nerve the outermost layer is a combination of perineurium and loose epineurium which are both dense connective tissue containing collagen and elastic fibres, small blood vessels and a variable amount of fat. Guided by [29] we have modelled the nerve according to the following assumptions:

- Ringer’s solution has been considered like water (n = 1.329).
- The epineurium is connective tissue composed of ground substance (n = 1.345), lipids (n = 1.45) and tissue fibres (n = 1.43 fully hydrated) in variable proportions. Since the *Xenopus laevis*’ sciatic nerve presents an external surface made of loose epineurial tissue, we have assumed a high fluid content (40%), with the connective fibres making up 70% of the dry mass and the lipids 30% [30]. This gives us a value of the refractive index of the epineurium n = 1.4.
- It was not possible to obtain a specific value for the elastic fibres, so we have considered the value of the fibrils inside the human sclera (n = 1.41).
- Axons, in our simulations, are made of axoplasm and axonal membrane which consist of water, lipids and proteins (n = 1.5). Considering the percentages observed in [31] and a volume fraction of 0.2% for the membrane, we obtain the refractive index of the axons n = 1.338.
- Myelin is composed of 40% water (n = 1.3290), and the rest is 70 to 85% lipids and ∼ 15 to 30% proteins [32]. We considered a 70-30% proportion for fat and proteins obtaining a refractive index of 1.4.
- the endoneurial fluid has been considered to be like the cerebrospinal fluid (n = 1.335) since they perform the same physiological function and is reasonable to assume they have the same physical properties.

The values used for each different tissue type are reported in Table 1.

**Table 1:** Tissues refractive indexes used for the simulation. It was not possible to obtain all the values for the specific specimen considered (*Xenopus laevis*), therefore some assumptions (discussed in the text) have been made.

Ringer’s solution	Epineurium	Elastic fibres	Axons	Myelin	Endoneurium
1.3290	1.4	1.41	1.338	1.4	1.335

Since the time an electromagnetic wave needs to travel a few centimetres in air and water is on the nanosecond scale, it is possible to simulate the scattering change undergone by neurons by running different simulations changing discretely the value of the refractive index of the sample.

To obtain value for the backscattered light change, we have first developed a ray tracing model to study what kind of change in refractive index would result in a forward scattering change as observed in [10]. We have then taken that value as the relative change in refractive index for our simulations.

### 3.3 Post-processing

As mentioned earlier, we have decided to remove from the simulation domain the whole splitter/combiner part and to obtain the interference between the reference and sample arms using an external software (Matlab R2017a). The FDTD simulations output the amplitude of the electric field in a preselected point every ten time steps. For each of the saved steps the signals obtained from the nerve and mirror simulations are summed and squared to obtain the intensity interference pattern (S1 Video shows different stages of the process). The OCT signal is then computed as the envelope of the interference pattern. As this is a time domain simulation, each step in time corresponds to a spatial step and therefore the signal as a function of time can be converted in a signal as a function of tissue depth.

## 4 Results

Table 2 shows the parameters used in the simulations reported in this paper.

**Table 2:** Parameters used for the simulations.

Spatial step	Temporal step	Wavelength	Grid dimensions
Δ*x* = Δ*y* = 8.5*nm*	$\Delta t=S_{c}\cdot \frac{\Delta x}{c}=2\times 10^{-17}s$	λ_0_ = 850*nm* = 100Δ*x*	100 × 17*μm*^2^

The simulations have been run with the two pulse lengths shown in Fig 2 for both models. After being post-processed, the signal assumes its final form where—depending on the resolution—it is possible to distinguish the different interfaces.

**Fig 6:**
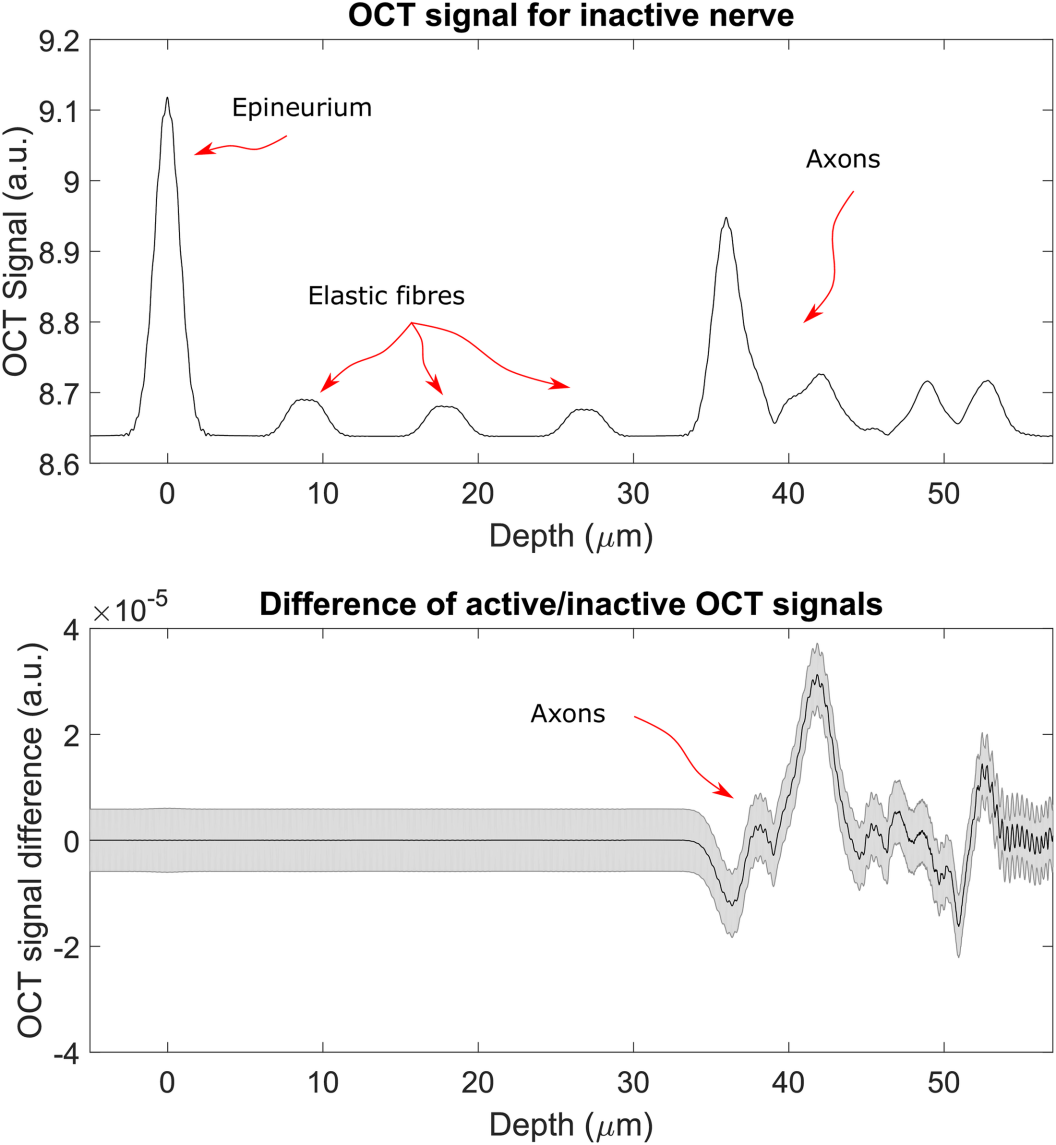
OCT signal for source with a *σ*_λ_ ∼ 76 nm. The top panel shows the intensity OCT signal in arbitrary units, in this figure it is possible to discern the various elements inside the tissues: epineurium, elastic fibres and axons are labelled. The bottom panel shows the difference between the intensity OCT signals obtained from the simulations for the inactive and active nerves normalised to the intensity of the incoming light. It is noticeable that, as expected, the change in signal is only due to the change in refractive index of the axons. The axons themselves are not discernible because the resolution is not high enough. The zero has been placed at the interface between water and epineurium and the grey shadowed area represents the poisson noise.

**Fig 7:**
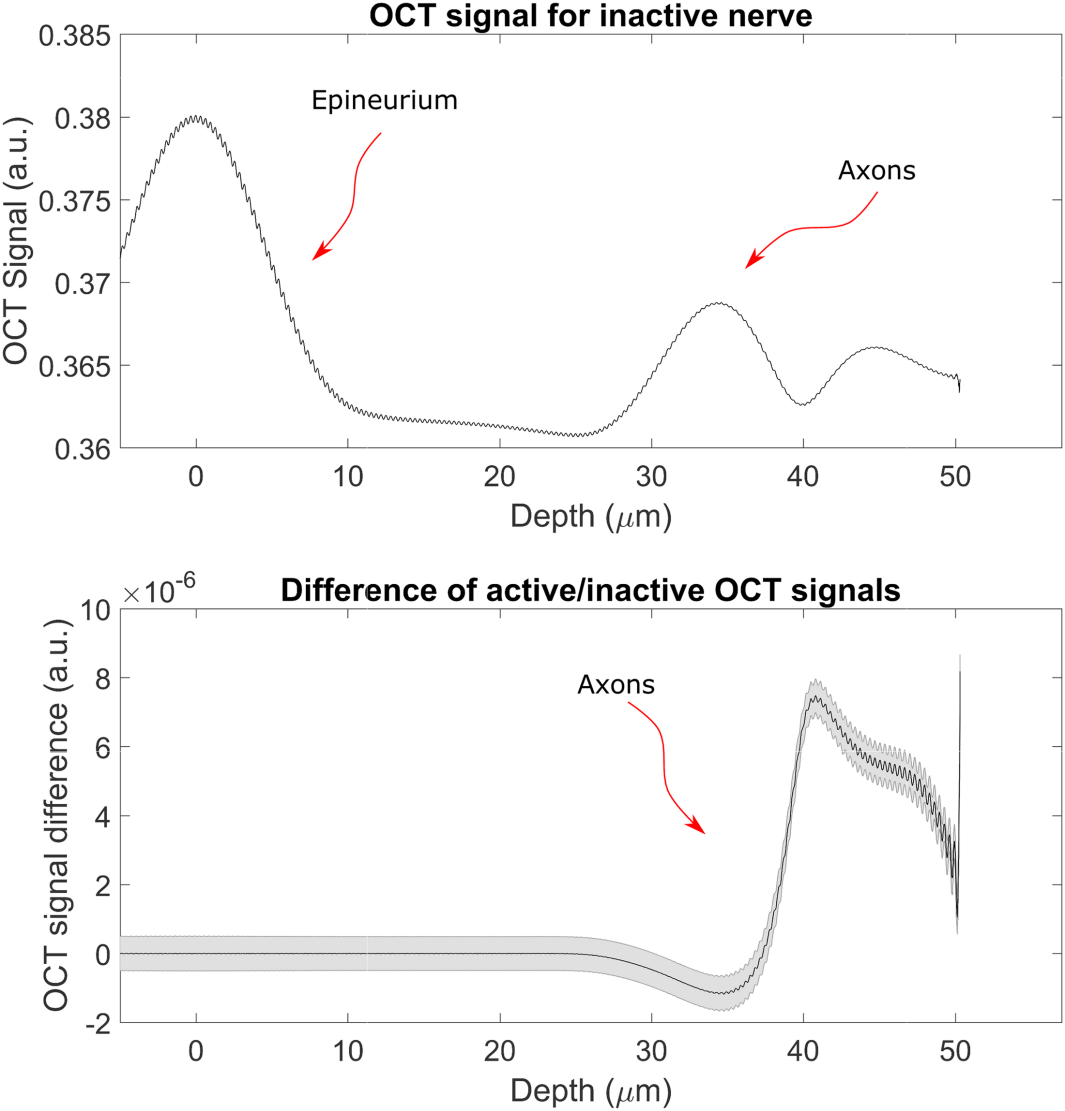
OCT signal for source with a *σ*_λ_ ∼ 13 nm. The top panel shows the intensity OCT signal in arbitrary units, in this it is not possible to discern all the various elements inside the tissues, as the resolution is not high enough. The bottom panel shows the difference between the intensity OCT signals obtained from the simulations for the inactive and active nerves normalised to the intensity of the incoming light. It is noticeable that, as expected, the change in signal is only due to the change in refractive index of the axons. The zero has been placed at the interface between water and epineurium and the grey shadowed area represents the poisson noise.

These simulations have given the expected results: in Fig 6, top panel, it is possible to see how the simulated OCT signal allows to distinguish between all the different regions of the nerve: first the interface between the Ringer’s solution layer and the nerve surface, the three elastic fibres inside the epineurium and the beginning of the nerve fibres region. In this last region the fibres cannot be singularly detected because of resolution limitations (both in the axial and transverse directions). In the bottom panel the signal obtained from the simulation representing the active nerve has been subtracted to the one obtained from the simulation representing inactive nerve. It is clearly visible that the signal from the part of the nerves which refractive index is common to both simulations cancels perfectly, leaving only a signal in the axon part. The same thing can be noticed in the bottom panel of Fig 7, but in this case the resolution of the scan is lower.

**Fig 8:**
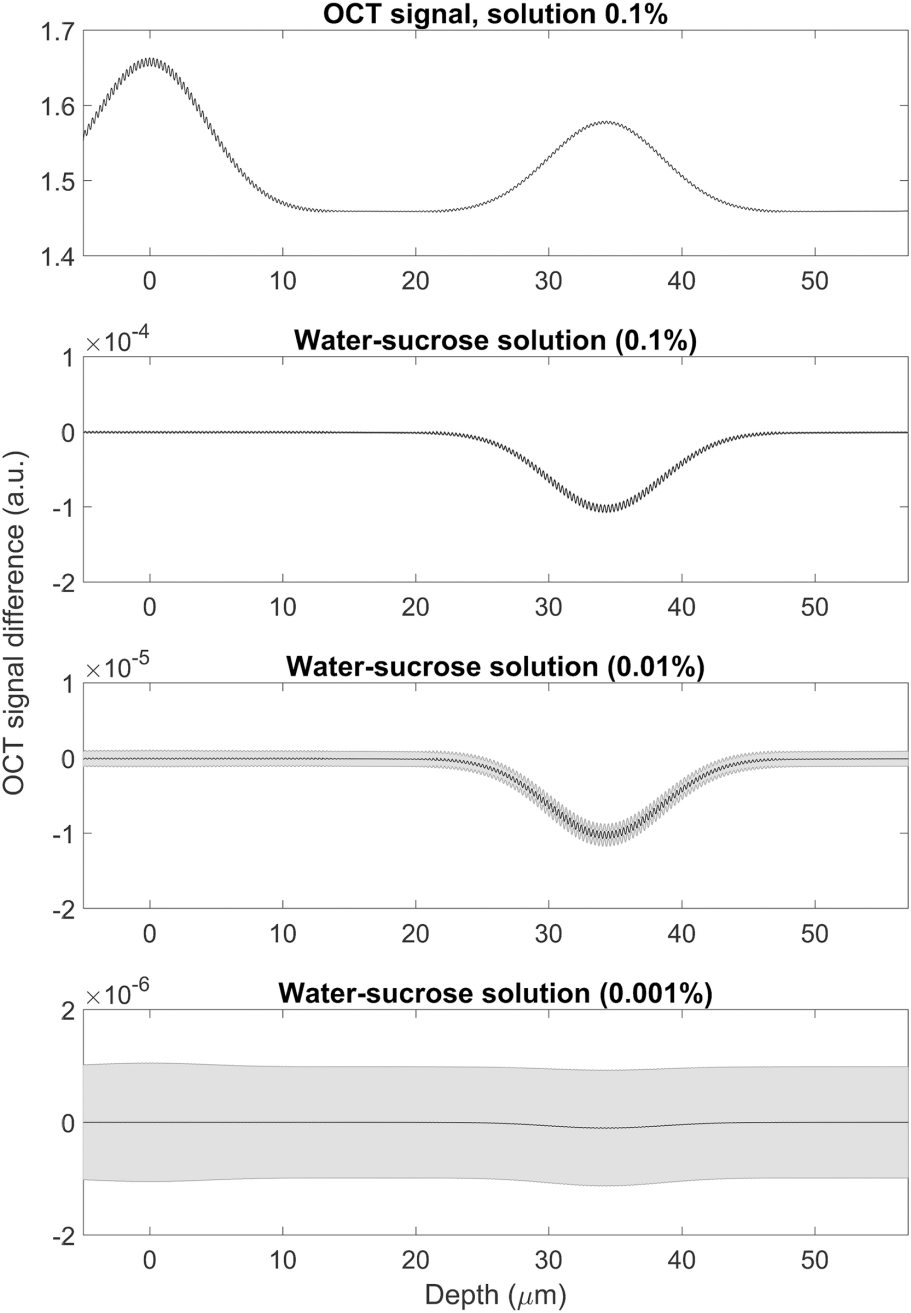
OCT signal for different concentrations of water-sucrose solutions and *σ*_λ_ ∼ 76 nm. Top panel: signal obtained for the glass rods model with a water-sucrose solution at 0.1%. Other panels: difference between the signal obtained from different concentrations (0.1%, 0.01% and 0.001% respectively) and pure water normalised to the intensity of the incoming light. The grey shadowed area represents the poisson noise.

The top panel of Fig 8 shows the OCT signal obtained for the glass rod model with a water-sucrose solution at 0.1%, the first peak being the result of the interface between water and glass and the second one between glass and solution. The other panels in the figure show the difference between the signal obtained from different concentrations (0.1%, 0.01% and 0.001% respectively) and pure water.

## 5 Discussion and conclusions

**Fig 9:**
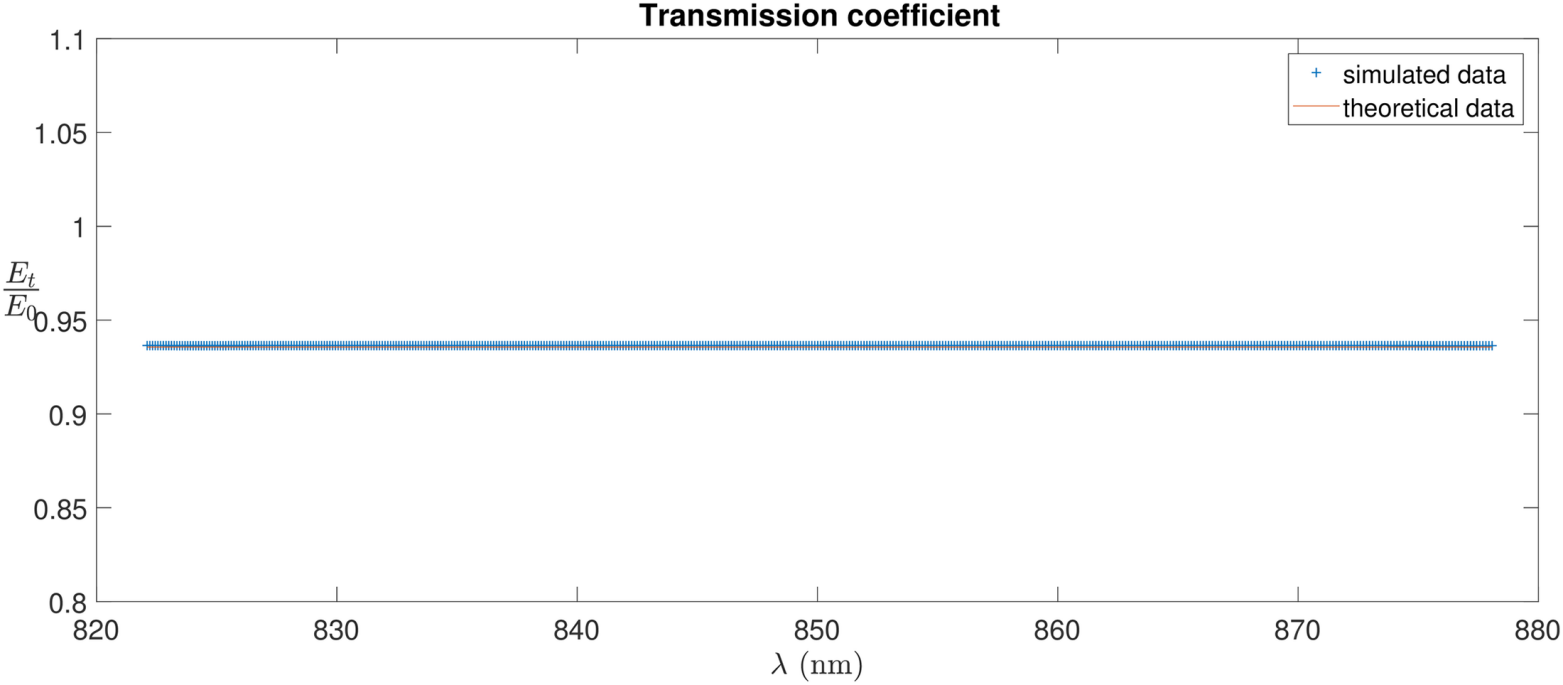
Transmission coefficient calculated theoretically using Fresnel equations (red line) and from the simulations (blue points).

A water-sucrose solution at the 0.01% appears to be a good approximation of the active nerve and an experiment has been planned to test the results obtained by the model before going into *ex-vivo* studies of the nerve. Fig 9 shows the transmission coefficient obtained for the interface water-glass in the simulations. The theoretical result, in absence of dispersion as given by Fresnel equations, is constant over the range of wavelengths considered; the simulated result is compatible with the theoretical one.

The data obtained from the simulations, after post processing, provide an A-Line OCT scan. To obtain a B-scan it would be possible to create multiple simulation domains corresponding to contiguous part of the tissue considered and run a simulation for each of them. While it is true that splitting the simulation domain—instead of having a domain that comprises the whole sample—would result in an underestimation of the noise coming from scattering inside the bigger domain, it is also true that for biological tissues at near infra-red wavelengths light experiences primarily forward scattering [33]. Since multiple simulations can be run at the same time, this would allow for a remarkable reduction in computational time even for 3D images.

Direct optical monitoring of the peripheral nerve activity by using a non-invasive, non-contact technique such as OCT will represent a major advancement in neurotechnologies. In this paper we are analysing the feasibility of a such approach and made some estimates about situations with non-ideal conditions. It is known that speckle noise plays a large role in OCT imaging and it is worth noticing that the FDTD simulation—by computing the electric and magnetic fields for each time and spatial steps—is intrinsically capable of taking the speckle noise into account. We have also introduced a general noise in the form of Poisson (shot) noise (the shadowed area in Figs 6, 7 and 8). Other sources of noise tend to be setup-dependant and therefore it is not be possible to include them in a general model. We can conclude by saying that the results in Figs 6, 7 and 8, demonstrate that Poisson noise shouldn’t prevent the use of optical coherence tomography to detect compound action potential.

## Supporting information

S1 Video Different stages of the post processing. In blue is the sample signal, in red the reference signal and in green the sum of the two.
(MP4)

S2 Video Colour map of the electric field amplitude at different points in time in the simulation. The first white vertical line represents the interface water-epineurium and the second the interface epineurium-nerve fibres.
(MP4)
