# Correction: Simulating optical coherence tomography for observing nerve activity: A finite difference time domain bi-dimensional model

**DOI:** 10.1371/journal.pone.0214688

**Published:** 2019-03-26

**Authors:** Francesca Troiani, Konstantin Nikolic, Timothy G. Constandinou

The following information is missing from the Funding statement: This project was supported by the European Research Council (ERC) Synergy Grant no. 319818.
